# Self-Perception of Dependence as an Indicator of Smartphone Addiction—Establishment of a Cutoff Point in the SPAI–Spain Inventory

**DOI:** 10.3390/ijerph17113838

**Published:** 2020-05-28

**Authors:** María Luisa Ballestar-Tarín, Conchín Simó-Sanz, Elena Chover-Sierra, Carlos Saus-Ortega, Carmen Casal-Angulo, Antonio Martínez-Sabater

**Affiliations:** 1 Nursing Department, Facultat d’Infermeria I Podologia, Universitat de València, 46010 Valencia, Spain; M.luisa.ballestar@uv.es (M.L.B.-T.); conchin.simo@uv.es (C.S.-S.); sausor@uv.es (C.S.-O.); m.carmen.casal@uv.es (C.C.-A.); Antonio.Martinez-Sabater@uv.es (A.M.-S.); 2 Hospital General, Universitario de Valencia, 46014 Valencia, Spain; 3 Nursing School La Fe, Universitario de Valencia, 46026 Valencia, Spain; 4 Emergencies Service, Extra-hospital, 46010 Valencia, Spain; 5 Hospital Clínico, Universitario de Valencia, 46010 Valencia, Spain

**Keywords:** smartphones, addiction, self-perceived addiction, ROC analysis, cutoff point, SPAI–Spain

## Abstract

Background: In recent years, the abusive use of the smartphone has reached a situation that could be considered pathological. In this sense, different instruments to assess this problematic use or addiction to the smartphone are used. One of these instruments is the Smartphone Addition Inventory (SPAI), which has been validated in the Spanish language (SPAI-Spain). The main difficulty of these scales is to establish a cut-off point that determines such mobile addiction. On the other hand, self-perception was used in different addictions as a predictor of the problem. Aim: The objective of this study was to establish the cut-off point in the scores of the SPAI-Spain, using as a reference the self-perception of addiction values. Methods: A receiver operating characteristics (ROC) analysis was carried out, establishing as the cut-off point the one that presented a higher value of Youden J, indicative of its sensitivity and specificity. Results: 2958 participants from the university community completed the SPAI–Spain questionnaire. Differences in SPAI–Spain scores were found among age groups and gender, even though not all of them were statistically significant. When using the self-perception of smartphone addiction as the benchmark value, a score of 44 was established as the cutting point of the SPAI-Spain questionnaire, with a Youden J corresponding to 0.416. Conclusions: The implementation of a cut-off point of the SPAI-Spain questionnaire makes it an instrument that allows early identification of those individuals at risk of addiction, as well as the establishment of preventive and/or intervention measures.

## 1. Introduction

In the Diagnostic and Statistical Manual of Mental Disorders (DSM–V), the term behavioral addiction is introduced, with the gaming disorder as the only category [1]. It is admitted, therefore, that the “pathological gambling” is an addictive disorder and not a disorder of impulse control, as it was previously classified [2,3]. The World Health Organization (WHO) included gambling and gaming disorder in the International Classification of Diseases (ICD) 11, suggesting that behavioral addictions share some common ground with substance use disorders [4]. Besides, the American Psychiatric Association (APA) recognizes that reward systems are activated in behavioral addictions, and similar behavioral symptoms occur in clinical conditions caused by substance use [1]. This is an open door to other behavioral addictions such as sex, shopping, food, work, physical exercise, mobile, or technologies [5]. However, the DSM–V only points out a small reference to the existence of these excessive behaviors, calling them behavioral syndromes. They are supposed not to have a solid base to be considered as mental disorders [1]. Despite this, many researchers suggest the addictive nature of these behaviors, although they do not share some features with substances’ addiction [4,6,7].

This study focuses on the use of the smartphone, to which authors such as Flores et al. [7] catalog it as the consequence of the new “society of autism.” The increase in connectivity in different areas of life has led to a behavioral change in people. Moreover, the excessive use of network technology can lead to physical, mental, and social problems [8]. This symptomatology is assimilated to substances addiction, giving rise to the lack of control of impulses, dependence, craving, anxiety, interferences in daily life, in the dream, and/or in personal relationships, among other symptoms [9]. Hence, currently, the problematic use of technologies is considered a social issue, with adolescents and young adults constituting the largest risk group [10,11,12,13].

It must be said that adolescence is a period when it is more likely to create dependence on the mobile phone due to the device’s almost irresistible characteristics, for instance: autonomy, freedom, identity, social prestige, and entertainment [14]. Although it is true, authors such as Lu et al. [15] suggest that the psychological dependence and abuse created with some of its functionalities are not something exclusive to adolescents. Nevertheless, some other studies indicate that this dependence could affect the adult population [16,17,18,19].

Some authors such as De-Sola et al. [20] and Lin [21] have already echoed the need for more studies in other population groups and higher age ranges because there is a lack of awareness about how this device works in them.

On top of that, the difference in use based on gender is another aspect that was analyzed regarding the use of the smartphone. Most studies identify that women use the smartphone much more, and they even have higher levels of addiction [19,22]. Besides, some authors speak of different patterns of use according to gender. Women use the smartphone as an instrument to communicate with others; in contrast, men use it as an instrument for accessing the Internet or gaming [22,23].

Additionally, authors such as Billieux consider the “problematic mobile phones’ use” (PMPU) as an inability to regulate the use of mobile phones, which eventually leads to negative consequences in daily life [24]. Studies carried out in Spain find percentages of people with PMPU that vary between 7.99% and 12.5% using different scales [25]. To this end, three different ways were established to define the problematic use of the mobile with its three respective behavior patterns: addictive pattern, antisocial pattern, and risk use pattern. It is necessary to know through validated instruments and semi-structured interviews three aspects of the individual to be catalogued as a user with a real problem: the user profile, the actual use of the device, and the type of problematic use [26]. To achieve the objective of establishing diagnostic criteria for behavioral addictions, it is also necessary to develop reliable and valid measures of these behaviors and regularly assess their psychometric properties, especially because the technological and social trends related to these behaviors change rapidly [8]. Diverse authors designed several instruments to evaluate this problematic use of the mobile phone [25], and even different diagnostic criteria were established for smartphone addiction [27].

Aside from these criteria, self-perception plays an important role in this work. Various authors defined this perception as the process by which people infer their attitudes from their behavior and which refers to the set of evaluations that a person has regarding their abilities [28]. In other words, it is the mental image of oneself. Each person creates it from his or her experiences and needs, and this self-perception is mediated by the circumstances that surround the individual. Escamilla, Córdoba, and Campos [29] consider it a dynamic reality that changes with experience, integrating new data and information, and develops according to social experiences.

In several studies, validated scales asking participants to express their perception regarding the risk indicated or perceived by them are used [30,31]. It is important to emphasize that the degree of problem recognition that anyone has can be related to the valence of the content of their scheme related to consumption [32]. Therefore, self-perception is widely used in different studies as an indicator of disease. In this sense, some factors for it were evaluated, such as the association of self-perception with the dependence on text messages [8], the use/addiction to pornography and personality factors [33], the reasons and barriers in the search for help in gambling problems [34], risk of committing violence [31], alcohol dependence [32,35], and predictors of change in alcohol consumption habits [36]. Even the self-perception of physical health is related as a predictive factor in the use of health services [37] or the degree of participation in endurance sports and self-reported data on self-image, physical and psychological health, and style of life in general [30]. Going further, in other studies, the variable self-perception of health status proves to be a criterion that is strongly related to the presence of chronic diseases [38].

Consequently, this study was performed to establish the cut-off point in the scores of the SPAI–Spain scale [39] related to the self-perceived addiction score indicated by the participants. Besides, the intention is to verify that this criterion variable is better than other measures of dependence, such as the mobile’s dedication time.

It is worth highlighting that SPAI is a smartphone addiction screening tool for the adult population, which is already proven to be valid and reliable by research carried out in different countries (China, Italy, Turkey, and Brazil) [40,41,42,43]. Its main advantage is its ability to assess aspects as important in other addictions such as compulsive behavior, functional impairment, withdrawal, and tolerance. The authors selected this instrument for its validation in Spanish [39] after carrying out a review of different instruments used to assess mobile addiction [25].

An exploratory study was thereby carried out to establish a cutoff point that allows discrimination between addicted and nonaddicted individuals. Our starting hypothesis is that dependency self-perception is a good indicator of the level of mobile addiction. Therefore, it will be an appropriate criterion to establish this cut-off point.

## 2. Materials and Methods

### 2.1. Design

It is a cross-sectional observational study. The data collection instrument (in online format) was distributed among university students by using mailing lists from the University of Valencia’s central services as a mean of dissemination after obtaining the permission of the University Rector’s office. Data were collected from 5 to 28 April 2017.

The University of Valencia postgraduate studies ethics committee reviewed and approved this study. All the participants gave their written consent to their voluntary and anonymous involvement before completing the inventory.

The inclusion criteria were as follows: age over 17 years old, having a smartphone, and being a student of any discipline at the University of Valencia. The participants were selected using nonprobabilistic convenience sampling. Although the selection of the participants was made by convenience sampling, it must be said that it reflected the reality of the university population.

### 2.2. Data Collection Instrument

The instrument designed allowed us to collect information about the sociodemographic characteristics of the population, patterns of mobile phone use, and the subjective perception of smartphone dependence, by using a numerical scale (1–10); the SPAI–Spain inventory used for the analysis of smartphone addiction was also included.

The SPAI–Spain instrument [39] shows adequate indices of internal consistency (α = 0.94) and consists of 22 items, four less than its original version [41]. Each item is answered by a Likert scale ranging from 1 (strongly agree) to 4 (strongly disagree), obtaining a global score between 22 and 88. The higher the score obtained, the higher the degree of addiction. The original SPAI scale [41] obtained a global Cronbach’s alpha of 0.949. Each of its corresponding factors, compulsive behavior, functional impairment, abstinence, and tolerance, obtained the following Cronbach’s alpha indexes = 0.856, 0.888, 0.855, and 0.712, respectively. The overall Cronbach’s alpha indexes of the SPAI–Spain version [39] were 0.95 for the global scale and 0.87, 0.88, 0.81, and 0.72 for each of its factors.

### 2.3. Data Analysis

Receiver operating characteristics (ROC) analysis was conducted to examine the diagnostic efficacy of the SPAI–Spain for smartphone addiction by using the area under the ROC curve (AUC). Through this analysis, the probability of correctly classifying a subject as an “achiever” of a specific characteristic (sensitivity of the test) was compared with the probability of classifying as “achiever” somebody who was not (1-specificity). This analysis leads to obtaining an optimal cutoff point, from which it was possible to correctly classify the most significant number of subjects [44].

Thus, to obtain a cut-off score of the SPAI–Spain, two variables that measure dependence on the smartphone, self-perception of the dependency and hours of dedication, were used as criterion variables. These two variables were chosen because both obtained high correlations with the final score of the SPAI–Spain, Spearman’s r 0.595, and 0.447, respectively.

Self-perception of dependency was evaluated using a Visual Analogical Scale (VAS). The subject was asked to rate their level of dependence from 0 to 10, with 0 being “I do not consider myself dependent on the smartphone at all” and 10 “I consider myself totally dependent on the smartphone.” We decided to use a VAS scale to evaluate dependence, in a similar way as this type of scales are used for assess pain levels, thereby being able to evaluate dependence objectively.

The phone usage time was evaluated by asking the subject the number of hours he or she spent using the smartphone daily (less than two hours, between two and four hours, or more than four hours).

Both criterion variables were dichotomized to conduct ROC analysis, considering as “dependent” those subjects who indicated scores between 8 and 10 in the smartphone’s dependence self-perception or those who used their phone 4 h or more a day. “Not dependent” subjects were those with scores lower than 8 in the variable smartphone’s dependence self-perception or those who used it less than 4 h a day.

The sensitivity, specificity, and Youden’s J were calculated for each SPAI–Spain score. The cutoff point for the SPAI–Spain was optimal for smartphone dependence diagnosis when the score was accompanied by the higher Youden’s J.

Youden’s J is the maximum vertical distance from the ROC curve to the line between (0, 0) and (1, 1). When sensitivity and specificity are equally weighted, the optimal cutoff point is the point with the highest value of J, calculated according to the formula (sensitivity + specificity – 1) [45].

Subsequently, the percentage of false negatives and true positives was assessed using a cross-tabulated table to determine the best criterion variable. Diagnostic accuracy achieved at each score was also calculated.

Analyses were carried out using SPSS Version 24.0. Armonk, NY: IBM Corp. and Microsoft Excel 2010 spreadsheets for Windows.

## 3. Results

### 3.1. Characteristics of the Studied Population

The SPAI–Spain questionnaire was completed by 2,958 participants whose sociodemographic characteristics, as well as the profile of use of the smartphone, are shown in Table 1.

### 3.2. ROC Analysis Results

**Figure 1:**
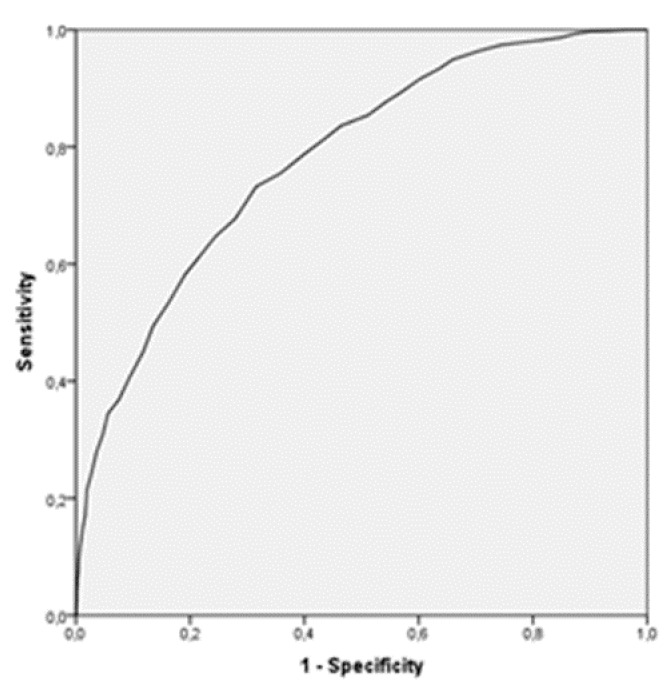
Receiver operating characteristics (ROC) curve. Criterion variable: self-dependence perception.

**Figure 2:**
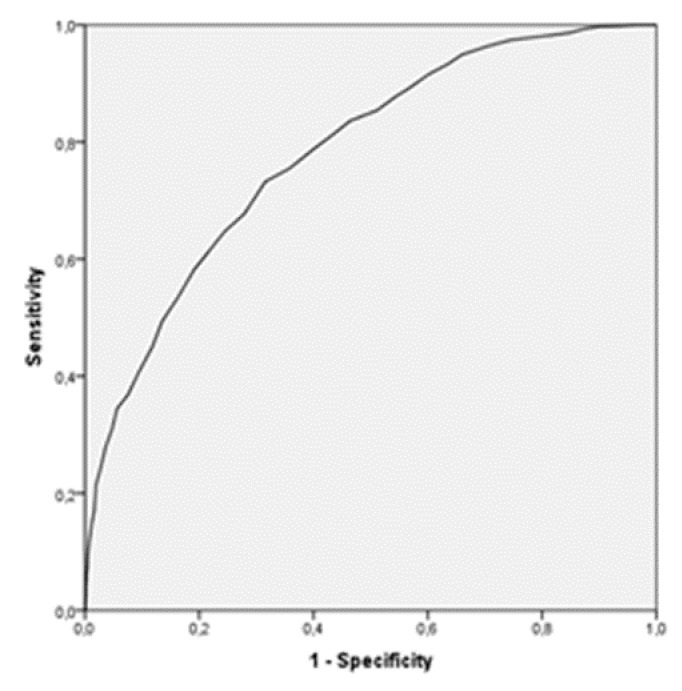
ROC curve. Criterion variable: hours of dedication.

The area under the curves is 0.78 [0.763–0.798] by using as a criterion variable the perception of dependence, and 0.699 [0.679–0.719] with the variable dedication time (in hours), as shown in Figure 1 and Figure 2, respectively.

### 3.3. SPAI–Spain Cutoff Score Determination

In order to identify the most suitable cutoff point, the sensitivity and specificity corresponding to each of the scores of the questionnaire for the variable dependence perception and dedication hours, as well as the Youden index, were calculated. It was established that the cutoff point would be the SPAI–Spain score corresponding to the highest value of the Youden index. In the case of dependence self-perception, it was established at score 44 (corresponding to a Youden’s J of 0.416). In contrast, in the case of the variable hours of dedication, the cut-off point was established at the value 42 (which corresponds to a Youden’s J value of 0.278).

In the supplementary files, we included detailed tables with sensitivity, specificity, positive and negative predictive values, diagnostic accuracy, and Youden’s J values for each SPAI–Spain score, according to both external criteria (dependence self-perception and dedication hours).

From these cut-off points identified according to the two established criteria, the percentage of subjects who would be adequately classified with each of said cutoff points was calculated. These results are shown in Table 2.

Although the cutoff point is stricter using the hours of dedication as a criterion variable, the percentage of true positives (dependent–addicts) is higher in the case of the variable self-perception of dependence (73.2% versus 71.8%). Likewise, the percentage of false negatives is higher by using as the variable self-perception of dependence rather than hours of dedication (68.4% versus 56.0%). Subsequently, the score 44 in SPAI–Spain is considered as its cutoff point. It is the one obtained when considering the subjective self-perception of dependence as a criterion since it allows us to classify better both the nondependent subjects and the dependent subjects.

Once the score of 44 (as the cutoff point of the questionnaire) was determined, the percentages of subjects that would be considered as dependent were analyzed, based on characteristics such as the participants’ gender and age. These results are shown in Table 3 and Table 4.

This analysis of the differences by using the chi-square test allowed us to conclude that there were no statistically significant differences between men and women (chi-square = 2.85 degrees of freedom (df) = 1; *p* = 0.09). However, some significant differences were perceived between age groups (chi-square = 106.68; df = 2; *p* < 0.001).

## 4. Discussion

In this research, we set out to establish the cutoff point of the SPAI scale validated in Spanish (SPAI–Spain), establishing self-perceived addiction as an external criterion.

The perceived addiction to the smartphone refers to the propensity of a person to report feelings of deregulation and a compulsive device use. In this situation, the focus is on the subjective assessment instead of the objectively measured behaviors, and the focus is on perception, which can be considered as a relevant clinical construct and predicts levels of psychological distress [33].

For this reason, it is understood that the self-perception of dependence on the smartphone, evaluated with the SPAI–Spain scale, can also be a predictor of this addiction. Thus, we divided subjects into “dependent or not dependent“ according to their subjective perception, which we have considered an optimal parameter to establish a cutoff point in the SPAI–Spain instrument that could allow researchers to identify those subjects with smartphone addiction. This self-perception of mobile dependence was also measured in other research, such as is this European study performed in 2017. In the study, it emerged that young people in southern Europe (including the Spanish) showed the highest phone usage time, being a predictor of their dependency levels, measured in this case with the Problematic Mobile Phone Use Questionnaire (PMPUQ) [46].

In the validation study of the SPAI–Spain version from which this work for the determination of a cut-off point arises [46], we set out to analyze a series of variables that would serve as comparison criteria with the results of the questionnaire. Among these collected data, we find a subjective criterion, such as the self-perception of mobile addiction, and an objective criterion, such as the number of hours an individual uses the mobile phone. For this reason, we tried to use both measures to establish the cutoff point, finding later that the subjective perception of dependence is a criterion that allows us to predict addiction better than the time the smartphone is used.

The methodology used to establish this cutoff point was based on the determination of the AUC in the ROC curve. Subsequently, the sensitivity (capability to detect addicted individuals), the specificity (capability to detect nonaddicted individuals), and the Youden index for each of the cut points were analyzed, choosing as cutoff that with a higher Youden index [44,47]. This methodology was used in other works in which the objective was to establish this cutoff point from which to establish a classification of the population based on the presence or absence of a specific characteristic, evaluated through any instrument [47,48,49].

When the self-perception of dependence on the smartphone was used as an external criterion, better diagnostic accuracy values were obtained than when using the criterion of daily hours of smartphone use. Therefore, it also points out that self-perception of dependence was a better indicator than usage hours. Thus, considering that the cutoff point based on the self-perception criterion was the one which allowed us to classify the participants in the study more adequately, it was decided to establish the value 44, which was the one with the highest Youden index (and therefore greater sensitivity).

Using this cut-off point, 23.67% of the participants in the study were classified as dependents to the smartphone. Furthermore, when considering their perception as a descriptor of this dependency, 32.24% of these participants considered themselves as a dependent. These results show how self-perception would be a good predictor of the level of dependence measured by the SPAI-Spain.

The establishment of this cut-off point allowed the identification of those subjects addicted or not addicted to the smartphone. This analysis was also established based on descriptive characteristics of the population, such as gender and age.

As for the analysis of the differences according to gender, this work found a higher percentage of women (53.88%) presented this addiction (although the differences were not statistically significant). In other studies that analyzed these differences, this trend of higher levels of smartphone addiction (or PMPU) in females continues.

In a study carried out in Madrid (Spain) among 1,328 young people, it was found that the estimated prevalence of cell phone dependency was 20% (26.1% in females, 13% in males) [50]. In addition to this, the study by Machado Kouri et al., developed in Brazil, identified female gender as a predictor of mobile addiction [42].

In a study carried out in Belgium and Finland by Lopez-Fernandez et al. [51], women also obtained higher scores in terms of smartphone dependence. The women who participated in the work carried out by Roberts et al. [52] reported using the mobile for longer hours and obtained higher scores on the instrument used to assess cell phone addiction. Also, Choliz et al. showed in two different studies [14,16] that females usually had a higher degree of dependency on mobile phones than did males.

In the case of the differences according to the age of the participants, this study showed statistically significant differences, by finding a higher percentage of mainly younger people who can be classified as addicts.

Most of the research about the excessive use of mobile phones focuses on young people. However, in studies that extend the age of the study population, this greater use or even dependence on mobile phones is also observed in younger people [18]. Thus, for example, the work of Machado Khouri et al. [53], in which subjects between 18 and 35 years of age participated, shows an inverse relationship between age and score in SPAI-BR, and that age between 18 and 25 is a predictive factor of addiction to the mobile. Smetaniuk, in a study with subjects between 18 and 75 years old [54], found significant differences in the score of the instrument used to assess mobile addiction between the different age groups, and the highest scores, indicators of higher addiction levels, were obtained by the youngest subjects, as observed in this work.

Several studies that allude to addiction to the Internet and mobile devices focused on the younger population (even at school age) and tried to identify factors that intervene in the development of this addiction. Some research showed that the younger a subject begins to use these devices, the more likely he or she is to develop addictive behaviors. This fact would explain the lower scores on the SPAI–Spain scale that were obtained in this work by older people who started using their devices at an older age.

It was not possible to compare the cut-off point established for the SPAI-Spain with the versions of the instrument in other languages since only two studies were found that proposed to establish this cutoff point. One of them is the study to develop the short version of the SPAI, with only ten items, instead of the 26 that compose the original instrument. In this study, some diagnostic criteria for smartphone addiction were defined. The cut-off point of the SPAI-SF as well as its capability to classify subjects as dependent and non-dependent, were established through ROC analysis based on the prior defined criteria [49]. There is another study that establishes a cutoff point that uses the Brazilian version of the instrument. In that study, which pinpoints the prevalence of smartphone addiction as an external benchmark for establishing the cut-off point, the researchers consider the 26 elements as dichotomous (yes/no) and establish the cutoff point in seven positive responses [42].

In the development of the original instrument the establishment of a cut-off point from which to define a subject as a dependent was not considered. Moreover, the two studies in which it has been established, the versions of SPAI (or the criteria to establish the cut-off point) are not similar, and also they have been adapted to different populations. All in all makes it difficult to compare the cut-off point established in this work with the other two cut-off points established for different versions of SPAI.

In other versions of the SPAI, such as the Turkish or the Italian one, the establishment of a cut-off point was not considered [40,43]. The study to validate the Iranian version of SPAI alludes to the need to establish a cut point in the instrument, in order to make it a diagnostic instrument, even this cut-off point was not established [54].

The establishment of a cutoff point in the SPAI–Spain instrument, to determine from what score a person could be considered as addicted, allows it to become a diagnostic instrument. An instrument useful to identify subjects with levels of dependence on smartphones and the establishment of measures to prevent/manage addresses this problem. It implies that in the daily practice of health professionals they can use an easy, simple, and manageable tool in the detection of mobile addiction. At the same time, it allows the screening of the general population in the existence of a disorder such as addiction to mobile phones, and thus it can establish therapeutic interventions. On the other hand, it is essential to assess the feeling of self-perception of people regarding their addiction, which will allow their involvement in these therapeutic interventions.

As other researchers claim, problematic mobile use is an evolving public health concern that requires more study to determine the boundary between helpful and harmful technology use [12]. The determination of a risk score can allow future establishment of mobile dependence prevalence studies, as well as the establishment of early detection programs and adequate treatment of this current public health problem.

The information provided by the SPAI–Spain instrument can be highly relevant as a support for researchers and clinicians in the diagnosis and treatment of problematic/addictive use of the smartphone in the adult population. It could be included among the instruments used for evaluating addictive disorders, as well as other tools such as the Münchner Alkoholismus Test (MALT) for the detection of problematic alcohol consumption or the Fagerström tobacco dependence test. As it is a self-administered tool, brief and simple, it can be used in any field of research as a screening method.

Finally, we must not forget that the present work has a series of limitations that we would like to consider.

Regarding the methodology used to establish the cutoff point, although it is the most used for this purpose, we must not forget that the result will depend on the external criterion chosen as the definition of “dependency” (it has been self-perception in this case). Besides, the selection of another external criterion could lead to a different cut point. It must be considered the diversity of criteria, methodological approaches, and lack of conceptual delimitation (abuse, misuse, dependency, and addiction) that guided the various studies related to the PMPU (problematic mobile phone use).

The delimitation of the sample to a university setting could have influenced the high level of studies of the sample and the high percentage of women. It would be interesting to have randomized samples from other populations. The same number of male and female participants in each of the age groups was not recruited. Even so, the participants in our work show a faithful reflection of the population of students at the University of Valencia, with a higher percentage of women, which decreases as the participants’ age increases. Besides, we found a percentage of subjects over 45 years old and even over 60 years old, because in this university there is a study program specifically aimed at people over 65 years old.

The means used for the diffusion of the questionnaire and data collection (Internet and email) imply a self-selection bias of the individuals that must also be taken into account. This bias entails that people who use technology regularly and those who use their smartphone more frequently are more likely to have responded.

## 5. Conclusions

The statistical analysis performed allows the establishment of a cut-off value for the SPAI–SP scale at 44, based on the self-perception of addiction.

The cut-off point established for the SPAI-Spain presents adequate sensitivity and specificity values, as well as adequate diagnostic accuracy.

The establishment of its cut-off point allows the use of the scale as a diagnostic tool to improve the detection and early treatment of addicted persons.

## Figures and Tables

**Table 1:** Characteristics of the studied population.

	Mean	SD	*n*	%
**Age**	27.96	12.13		
18–25			1944	65.7
26–35			408	13.8
36–45			253	8.6
Over 46			353	11.9
**Gender**				
Men			1025	34.7
Women			1933	65.3
**Age When Started Using A Smartphone**	17.11	9.27		
**Smartphone’s Dedication Hours**				
<4 (Nondependent)			2062	69.7
≥4 (Dependent)			896	30.3
**Smartphone’s Dependence Perception**				
<8 (Nondependent)			2005	67.8
≥8 (Dependent)			953	32.2

SD: Standard Deviation; *n*: number of participants with this characteristic; %: percentage of participants with this characteristic.

**Table 2:** Participant’s classification according to their addiction level for the cutoff point for each criterion variable.

			**SPAI–Spain:**		
			**Cut-off point = 44**		
		Nondependent		Dependent	
		*n*	%	*n*	%
**Dependence Self-Perception**	Nondependent	1371	68.4	632	31.6
	Dependent	255	26.8	698	73.2
			**SPAI–Spain:**		
			**Cut-off point = 42**		
		Nondependent		Dependent	
		*n*	%	*n*	%
**Hours of Dedication**	Nondependent	1154	56.0	906	44.0
	Dependent	254	28.2	643	71.8

SPAI-Spain: SmartPhone Addiction Inventory Spanish Version; *n*: number of participants with this characteristic.

**Table 3:** Participant’s smartphone dependence according to the variable gender.

		Nondependent		Dependent	
		*N*	%	*N*	%
**Gender**	Female	1041	53.9	891	46.1
	Male	585	57.1	439	42.9

**Table 4:** Participant’s smartphone dependence according to the variable age group.

		Nondependent		Dependent	
		*N*	%	*N*	%
**Age Group**	18–25	956	49.2	988	50.8
	26–35	240	59.0	167	41.0
	36–45	157	62.1	96	37.9
	Over 46	273	77.6	79	22.4

## Supplementary Materials

The following are available online at https://www.mdpi.com/1660-4601/17/11/3838/s1, Table S1: Sensitivity, specificity, positive and negative predictive values, diagnostic accuracy and Youden Index of cut-off points in SPAI-SP considering self-perception of dependence, Table S2: Sensitivity, specificity, positive and negative predictive values, diagnostic accuracy, and Youden Index of cut-off points in SPAI-SP considering daily hours of smartphone use.
