# Supplementary material for: Self-Perception of Dependence as an Indicator of Smartphone Addiction—Establishment of a Cutoff Point in the SPAI–Spain Inventory
Source: Int J Environ Res Public Health. 2020 May 28;17(11):3838. doi: 10.3390/ijerph17113838 (PMC7312961; doi:10.3390/ijerph17113838)
Supplement: Supplementary file 1 [file ijerph-17-03838-s001.pdf]

Supplementary Material

Self-Perception of Dependence as an Indicator of Smartphone Addiction—Establishment of a Cutoff Point in the SPAI–Spain Inventory

María Luisa Ballestar-Tarín [1], Conchín Simó-Sanz [1,2], Elena Chover-Sierra [1,2,*], Carlos Saus-Ortega [1,3], María del Carmen Casal-Angulo [1,4] and Antonio Martínez-Sabater [1,5]

1 Nursing Department, Facultat d'Infermeria I Podologia, Universitat de València, 46010, Valencia, Spain; M.luisa.ballestar@uv.es (M.L.B.-T.); conchin.simo@uv.es (C.S.-S.); sausor@uv.es (C.S.-O.); m.carmen.casal@uv.es (M.d.C.C.-A.); Antonio.Martinez-Sabater@uv.es (A.M.-S.);
2 Hospital General, Universitario de Valencia, 46014 Valencia, Spain
3 Nursing School La Fe, Universitario de Valencia, 46026.Valencia, Spain; sausor@uv.es
4 Emergencies Service, Extra-hospital, 46010 Valencia, Spain
5 Hospital Clínico, Universitario de Valencia, 46010 Valencia, Spain
* Correspondence: elena.chover@uv.es; Tel.:+34-96-3864182

Received: 30 April 2020; Accepted: 25 May 2020; Published: date

Table 1. Sensitivity, specificity, positive and negative predictive values, diagnostic accuracy and Youden Index of cut-off points in SPAI-SP considering self-perception of dependence.

Score	Sensitivity	Specificity	PPV	NPV	DA	J (Youden)
23	1,000	0,00	1,00		32,2%	0,000
24	1,000	0,03	1,03	0,00	32,9%	0,029
25	0,999	0,05	1,05	0,02	33,3%	0,045
26	0,998	0,07	1,07	0,03	33,8%	0,069
27	0,997	0,10	1,11	0,03	34,5%	0,097
28	0,994	0,12	1,13	0,05	35%	0,118
29	0,986	0,15	1,16	0,09	35,6%	0,139
30	0,982	0,19	1,21	0,10	36,5%	0,168
31	0,978	0,22	1,26	0,10	37,5%	0,201
32	0,974	0,26	1,31	0,10	38,4%	0,230
33	0,963	0,30	1,37	0,12	39,4%	0,260
34	0,950	0,34	1,44	0,15	40,6%	0,289
35	0,933	0,37	1,47	0,18	41,2%	0,298
36	0,916	0,40	1,52	0,21	42%	0,314
37	0,892	0,43	1,57	0,25	42,8%	0,325
38	0,877	0,46	1,61	0,27	43,5%	0,334
39	0,854	0,49	1,67	0,30	44,3%	0,343
40	0,836	0,54	1,80	0,31	46,1%	0,372
41	0,815	0,56	1,86	0,33	47%	0,378
42	0,787	0,60	1,97	0,35	48,4%	0,388
43	0,754	0,64	2,11	0,38	50,1%	0,397
**44**	**0,732**	**0,68**	**2,32**	**0,39**	**52,5%**	**0,417**
45	0,677	0,72	2,43	0,45	53,6%	0,398
46	0,647	0,76	2,65	0,47	55,7%	0,403
47	0,611	0,79	2,84	0,50	57.5%	0,396
48	0,582	0,81	3,05	0,52	59,2%	0,391
49	0,533	0,84	3,30	0,56	61,1%	0,371
50	0,494	0,86	3,65	0,58	63,5%	0,359
51	0,451	0,88	3,81	0,62	64,5%	0,333
52	0,406	0,91	4,33	0,66	67,3%	0,312
53	0,368	0,92	4,89	0,68	69,9%	0,293
54	0,345	0,94	6,12	0,69	74,4%	0,289
55	0,308	0,95	6,57	0,73	75,8%	0,262
56	0,278	0,96	7,74	0,75	78,6%	0,242
57	0,241	0,97	8,95	0,78	81%	0,214
58	0,215	0,98	11,05	0,80	84%	0,196
59	0,172	0,98	10,45	0,84	83,2%	0,156
60	0,146	0,99	12,70	0,86	85,8%	0,134
61	0,128	0,99	14,25	0,88	87,1%	0,119
62	0,108	0,99	16,65	0,90	88,8%	0,102
63	0,089	1,00	17,87	0,92	89,5%	0,084
64	0,073	1,00	16,35	0,93	88,6%	0,069
65	0,062	1,00	20,67	0,94	90,8%	0,059
66	0,052	1,00	17,51	0,95	89,3%	0,049
67	0,042	1,00	21,02	0,96	90,9%	0,040
68	0,035	1,00	17,34	0,97	89,2%	0,033
69	0,028	1,00	14,19	0,97	87,1%	0,026
70	0,024	1,00	12,09	0,98	85,2%	0,022
71	0,019	1,00	12,61	0,98	85,7%	0,017
72	0,014	1,00	9,11	0,99	81,3%	0,012
73	0,008	1,00	8,41	0,99	80%	0,007
74	0,007	1,00	7,36	0,99	77,8%	0,006
75						-1,000
76	0,005	1,00	5,25	1,00	71,4%	0,004
77						-1,000
78	0,004	1,00	4,20	1,00	66,7%	0,003
79	0,003	1,00	3,15	1,00	60%	0,002
80	0,003	1,00		1,00	75%	0,003
81	0,002	1,00		1,00	66,7%	0,002
82	0,001	1,00		1,00	50%	0,001
83	0,000	1,00		1,00	50%	0,000

PPV: Positive predictive value; NPV: Negative predictive value; DA: Diagnostic accuracy.

Table 2. Sensitivity, specificity, positive and negative predictive values, diagnostic accuracy, and Youden Index of cut-off points in SPAI-SP considering daily hours of smartphone use.

Score	Sensitivity	Specificity	PPV	NPV	DA	J (Youden)
23	1,000	0,000	**1,00**		30,3%	0,000
24	1,000	0,029	**1,03**	**0,00**	30,9%	0,029
25	0,999	0,045	**1,05**	**0,02**	31,3%	0,044
26	0,996	0,068	**1,07**	**0,07**	31,7%	0,064
27	0,991	0,095	**1,10**	**0,09**	32,3%	0,086
28	0,982	0,116	**1,11**	**0,15**	32,6%	0,098
29	0,977	0,145	**1,14**	**0,16**	33,2%	0,121
30	0,970	0,176	**1,18**	**0,17**	33,9%	0,146
31	0,959	0,209	**1,21**	**0,20**	34,5%	0,168
32	0,948	0,239	**1,24**	**0,22**	35,1%	0,186
33	0,927	0,274	**1,28**	**0,26**	35,7%	0,201
34	0,906	0,313	**1,32**	**0,30**	36,4%	0,219
35	0,891	0,339	**1,35**	**0,32**	36,9%	0,229
36	0,874	0,371	**1,39**	**0,34**	37,7%	0,245
37	0,855	0,408	**1,44**	**0,36**	38,6%	0,263
38	0,838	0,431	**1,47**	**0,38**	39%	0,269
39	0,815	0,462	**1,51**	**0,40**	39,7%	0,277
40	0,775	0,499	**1,54**	**0,45**	40,2%	0,273
41	0,751	0,524	**1,58**	**0,47**	40,7%	0,275
42	0,718	0,560	**1,63**	**0,50**	41,5%	0,278
43	0,673	0,596	**1,67**	**0,55**	42%	0,269
44	0,634	0,630	**1,71**	**0,58**	42,7%	0,264
45	0,584	0,670	**1,77**	**0,62**	43,5%	0,254
46	0,549	0,701	**1,84**	**0,64**	44,4%	0,251
47	0,518	0,734	**1,95**	**0,66**	45,8%	0,252
48	0,491	0,758	**2,03**	**0,67**	46,9%	0,249
49	0,446	0,790	**2,13**	**0,70**	48,1%	0,237
50	0,412	0,819	**2,27**	**0,72**	49,7%	0,231
51	0,385	0,844	**2,46**	**0,73**	51,7%	0,229
52	0,343	0,870	**2,63**	**0,76**	53,4%	0,213
53	0,315	0,893	**2,95**	**0,77**	56,2%	0,208
54	0,288	0,911	**3,22**	**0,78**	58,4%	0,199
55	0,258	0,924	**3,38**	**0,80**	59,5%	0,182
56	0,225	0,934	**3,44**	**0,83**	59,9%	0,160
57	0,194	0,947	**3,64**	**0,85**	61,3%	0,141
58	0,165	0,953	**3,54**	**0,88**	60,7%	0,119
59	0,133	0,962	**3,51**	**0,90**	60,4%	0,095
60	0,109	0,969	**3,52**	**0,92**	60,5%	0,078
61	0,096	0,974	**3,66**	**0,93**	61,4%	0,070
62	0,079	0,978	**3,63**	**0,94**	61,2%	0,057
63	0,061	0,981	**3,16**	**0,96**	57,9%	0,042
64	0,050	0,983	**3,04**	**0,97**	57%	0,034
65	0,042	0,987	**3,24**	**0,97**	58,5%	0,029
66	0,037	0,989	**3,30**	**0,97**	58,9%	0,026
67	0,031	0,992	**4,02**	**0,98**	63,6%	0,023
68	0,026	0,993	**3,78**	**0,98**	62,2%	0,019
69	0,023	0,995	**4,83**	**0,98**	67,7%	0,019
70	0,021	0,996	**5,46**	**0,98**	70,4%	0,017
71	0,018	0,998	**7,36**	**0,98**	76,2%	0,015
72	0,012	0,998	**5,06**	**0,99**	68,8%	0,010
73	0,009	0,999	**9,20**	**0,99**	80%	0,008
74	0,008	0,999	**8,05**	**0,99**	77,8%	0,007
75						-1,000
76	0,006	0,999	**5,75**	**1,00**	71,4%	0,005
77						-1,000
78	0,004	0,999	**4,60**	**1,00**	66,7%	0,003
79	0,003	0,999	**3,45**	**1,00**	60%	0,002
80	0,002	0,999	**2,30**	**1,00**	50%	0,001
81	0,001	0,999	**1,15**	**1,00**	33,3%	0,000
82	0,000	0,999	**0,00**	**1,00**	0	-0,001
83	0,000	1,000		**1,00**	0	0,000

PPV: Positive predictive value; NPV: Negative predictive value; DA: Diagnostic accuracy.
